# Supplementary material for: An Aptamer against MNK1 for Non-Small Cell Lung Cancer Treatment
Source: Pharmaceutics. 2023 Apr 18;15(4):1273. doi: 10.3390/pharmaceutics15041273 (PMC10146192; doi:10.3390/pharmaceutics15041273)

## Slide 1
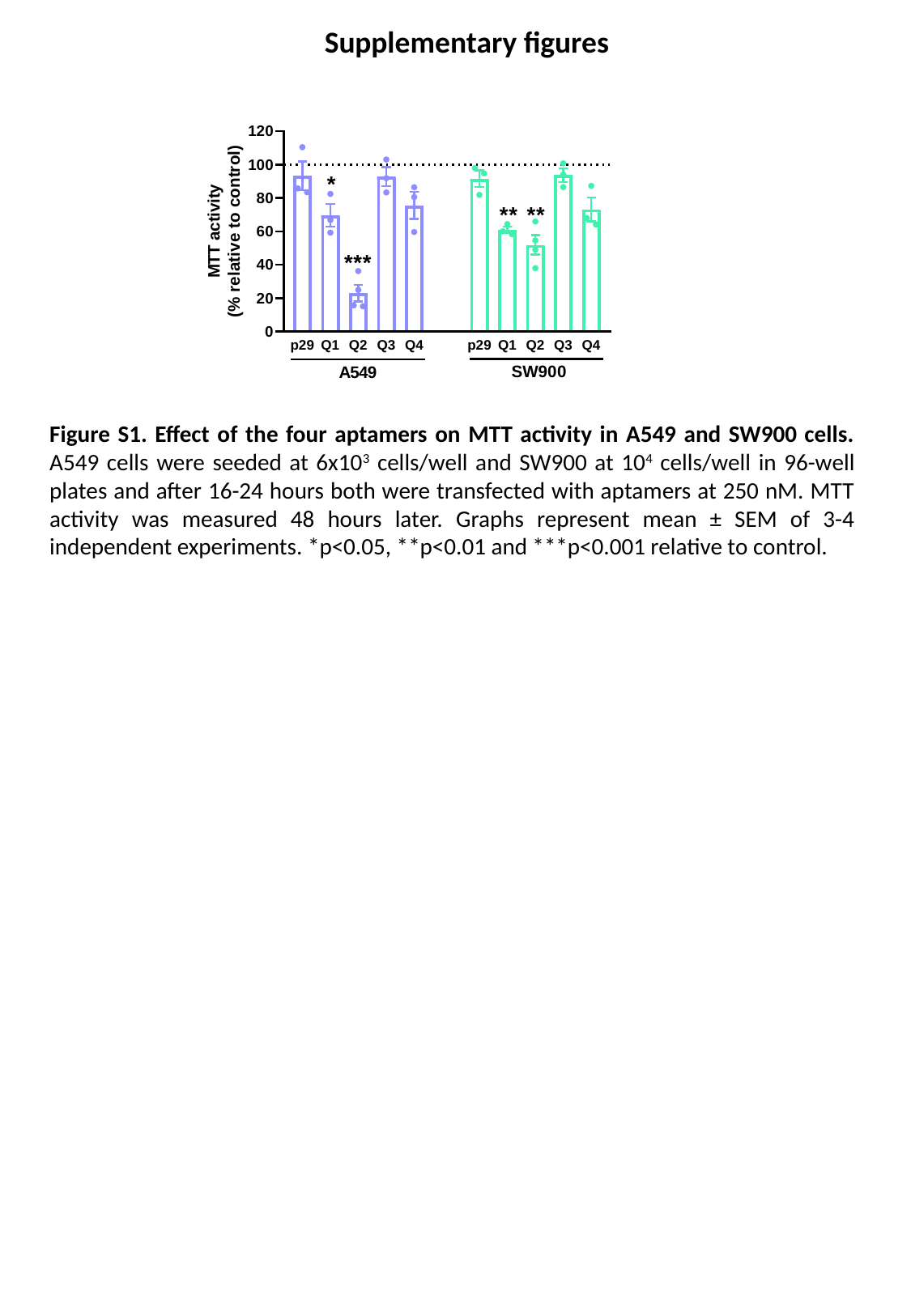

Supplementary figures
Figure S1. Effect of the four aptamers on MTT activity in A549 and SW900 cells. A549 cells were seeded at 6x10^3^ cells/well and SW900 at 104 cells/well in 96-well plates and after 16-24 hours both were transfected with aptamers at 250 nM. MTT activity was measured 48 hours later. Graphs represent mean ± SEM of 3-4 independent experiments. *p<0.05, **p<0.01 and ***p<0.001 relative to control.

## Slide 2
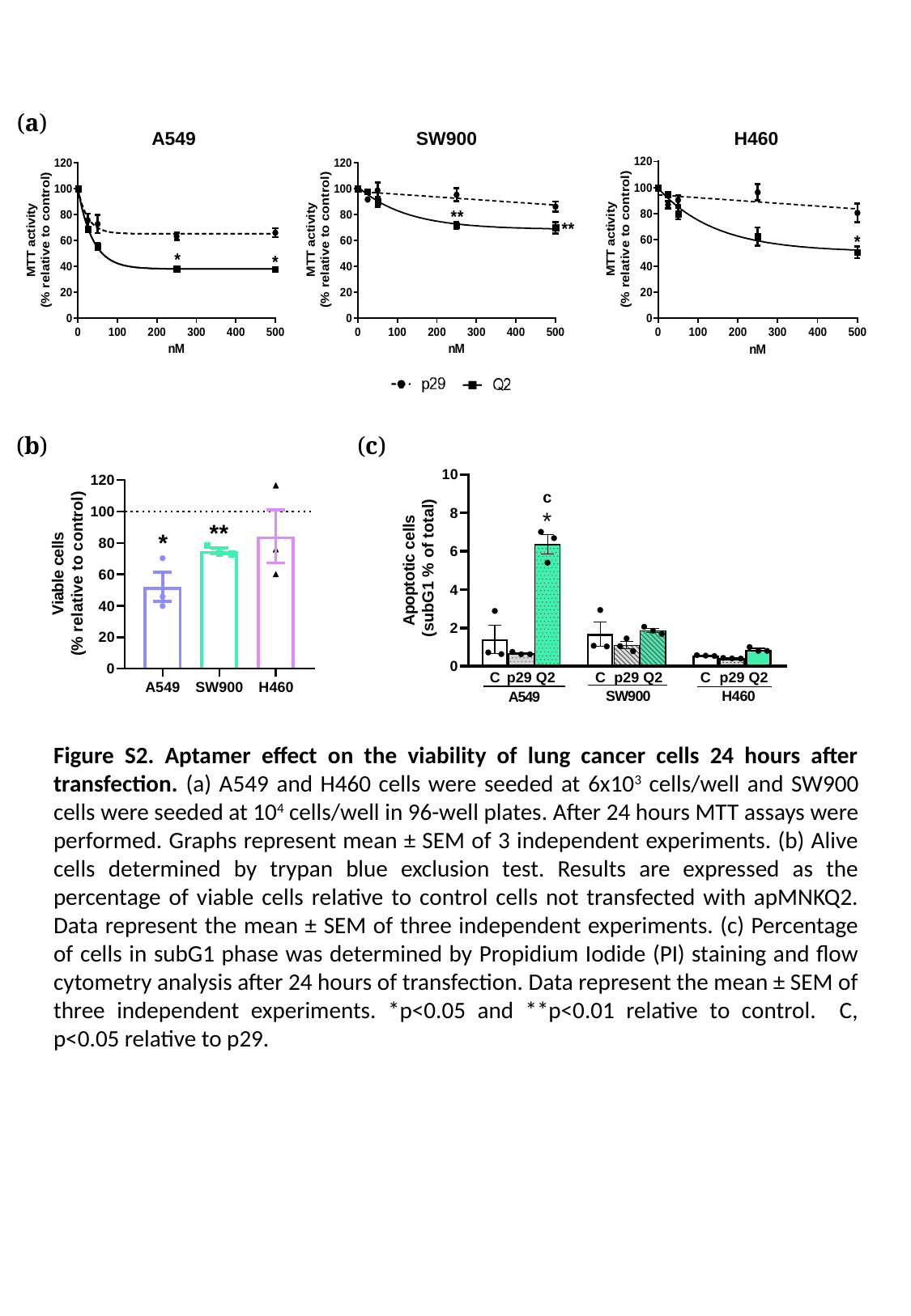

(a)
A549
SW900
H460
(b)
(c)
Figure S2. Aptamer effect on the viability of lung cancer cells 24 hours after transfection. (a) A549 and H460 cells were seeded at 6x10^3^ cells/well and SW900 cells were seeded at 104 cells/well in 96-well plates. After 24 hours MTT assays were performed. Graphs represent mean ± SEM of 3 independent experiments. (b) Alive cells determined by trypan blue exclusion test. Results are expressed as the percentage of viable cells relative to control cells not transfected with apMNKQ2. Data represent the mean ± SEM of three independent experiments. (c) Percentage of cells in subG1 phase was determined by Propidium Iodide (PI) staining and flow cytometry analysis after 24 hours of transfection. Data represent the mean ± SEM of three independent experiments. *p<0.05 and **p<0.01 relative to control. C, p<0.05 relative to p29.

## Slide 3
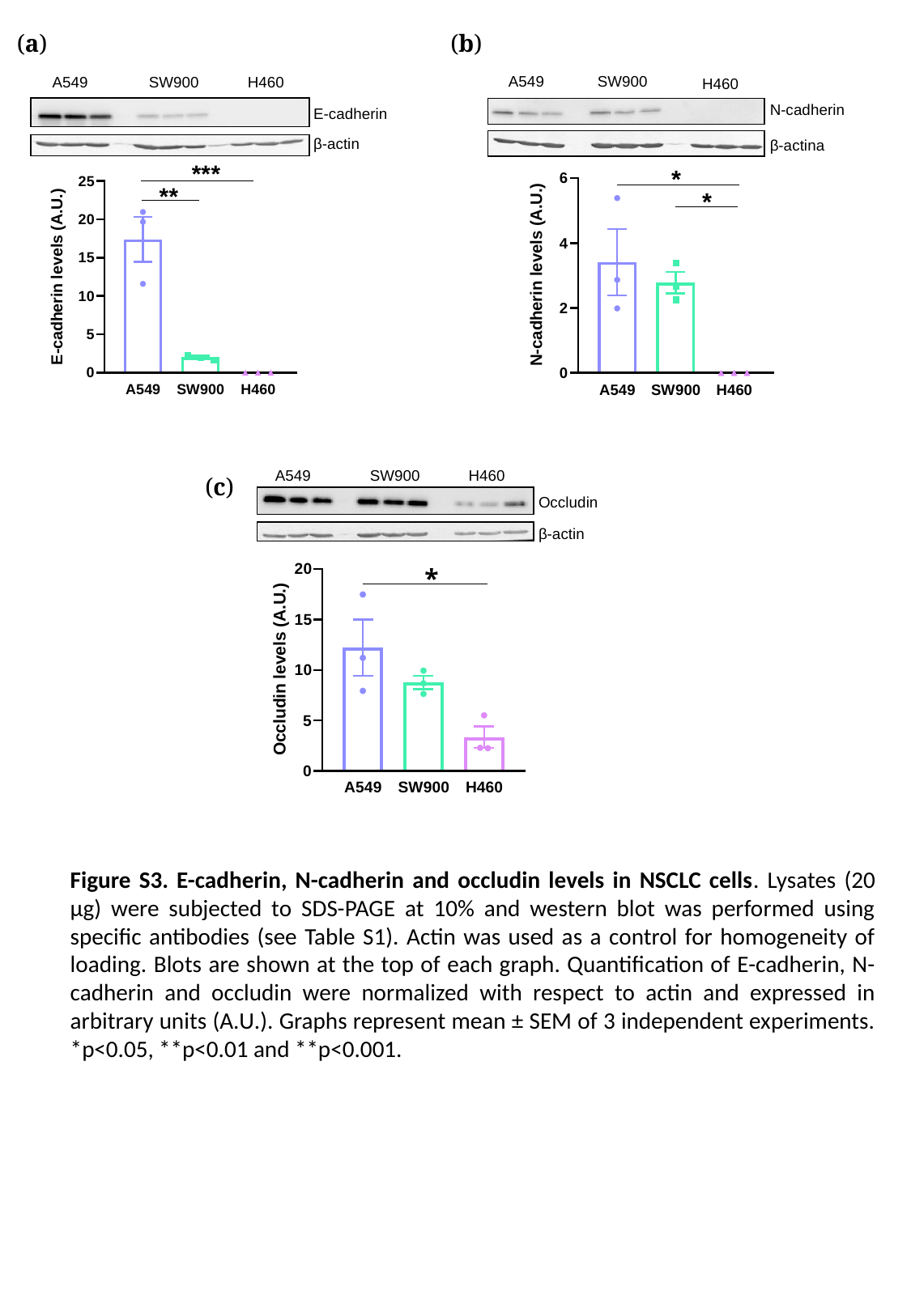

(a)
(b)
A549
SW900
H460
N-cadherin
β-actina
A549
SW900
H460
E-cadherin
β-actin
A549
SW900
H460
Occludin
β-actin
(c)
Figure S3. E-cadherin, N-cadherin and occludin levels in NSCLC cells. Lysates (20 µg) were subjected to SDS-PAGE at 10% and western blot was performed using specific antibodies (see Table S1). Actin was used as a control for homogeneity of loading. Blots are shown at the top of each graph. Quantification of E-cadherin, N-cadherin and occludin were normalized with respect to actin and expressed in arbitrary units (A.U.). Graphs represent mean ± SEM of 3 independent experiments. *p<0.05, **p<0.01 and **p<0.001.

## Slide 4
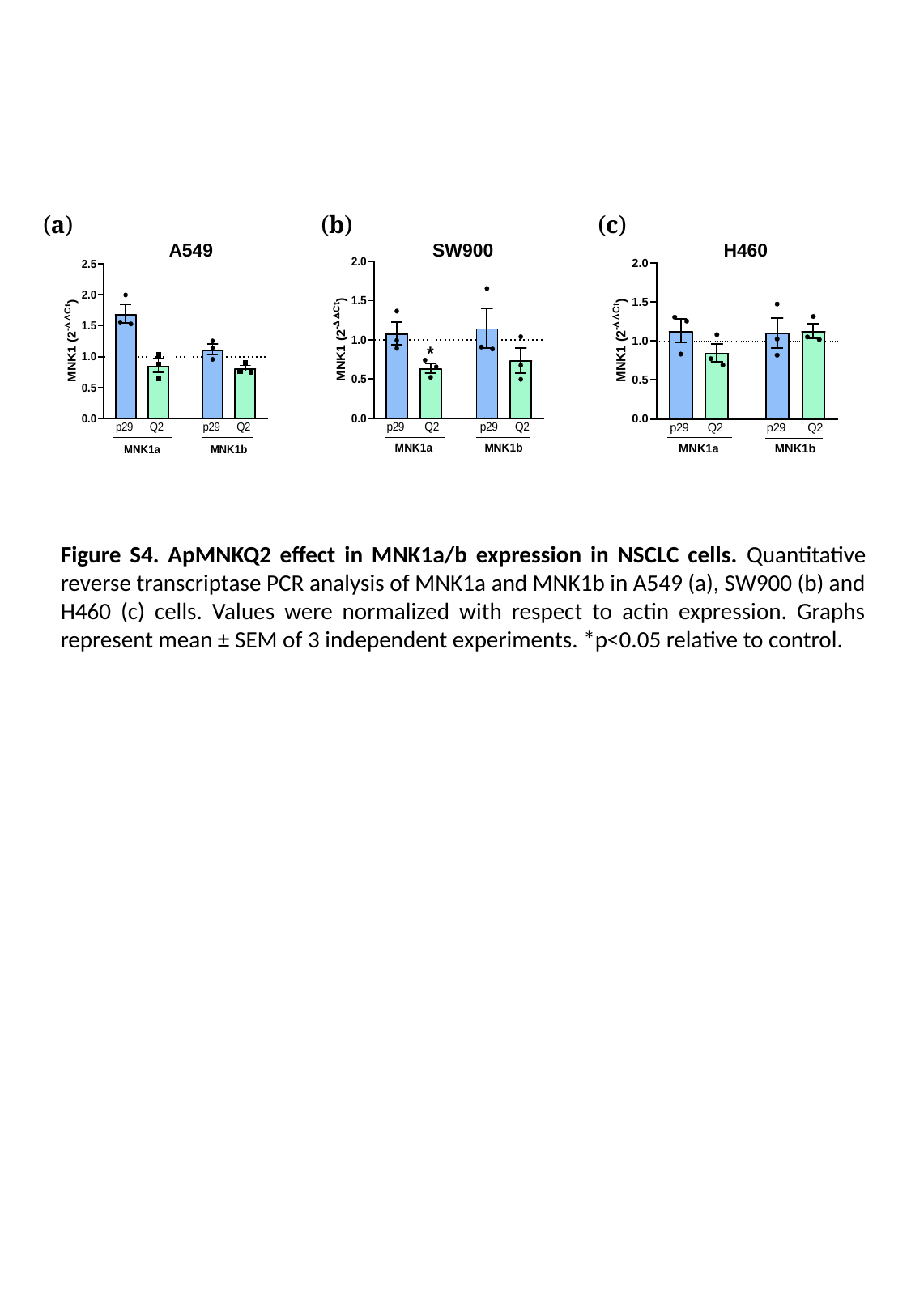

(a)
(b)
(c)
A549
SW900
H460
Figure S4. ApMNKQ2 effect in MNK1a/b expression in NSCLC cells. Quantitative reverse transcriptase PCR analysis of MNK1a and MNK1b in A549 (a), SW900 (b) and H460 (c) cells. Values were normalized with respect to actin expression. Graphs represent mean ± SEM of 3 independent experiments. *p<0.05 relative to control.

## Slide 5
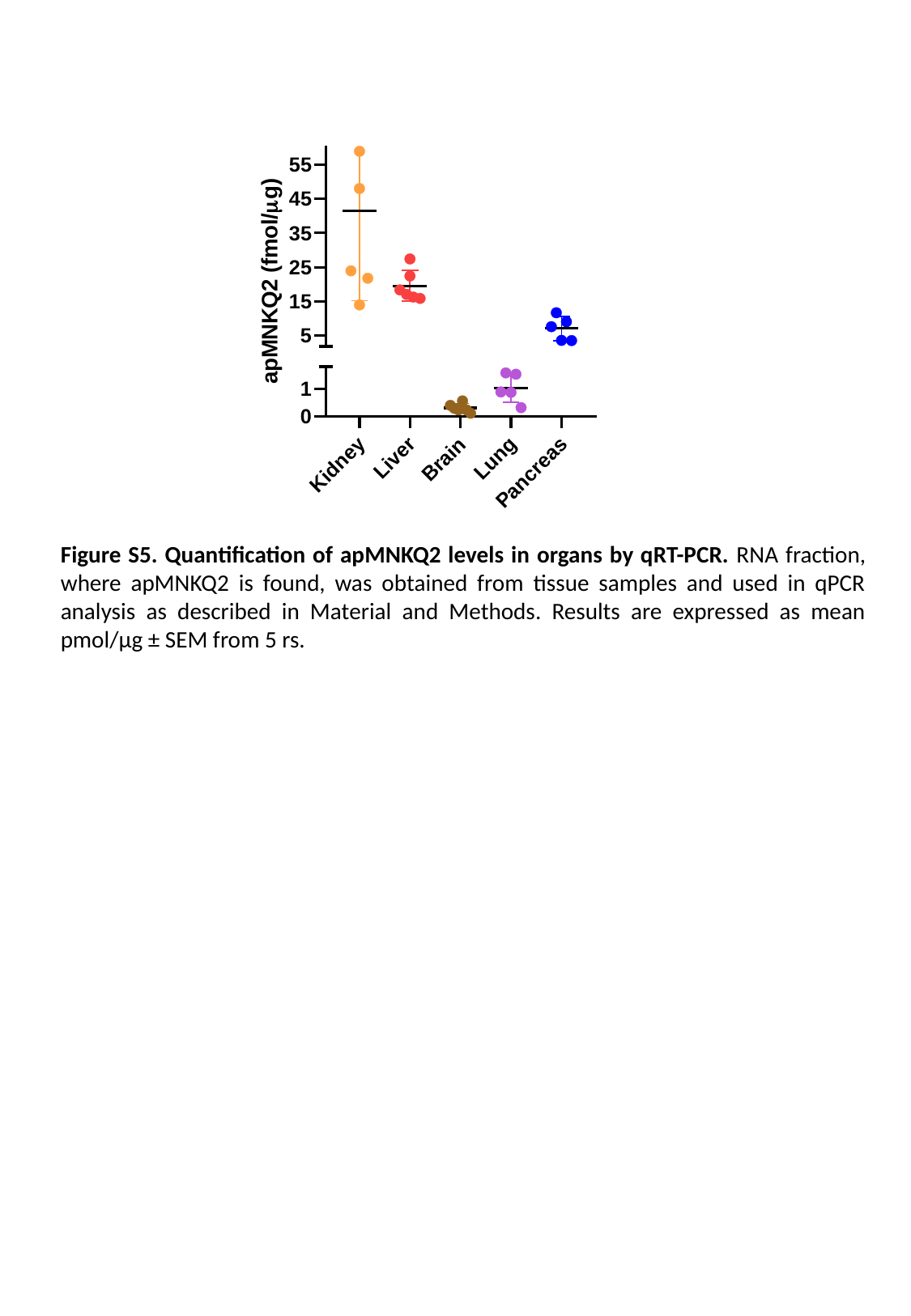

Figure S5. Quantification of apMNKQ2 levels in organs by qRT-PCR. RNA fraction, where apMNKQ2 is found, was obtained from tissue samples and used in qPCR analysis as described in Material and Methods. Results are expressed as mean pmol/µg ± SEM from 5 rs.

## Slide 6
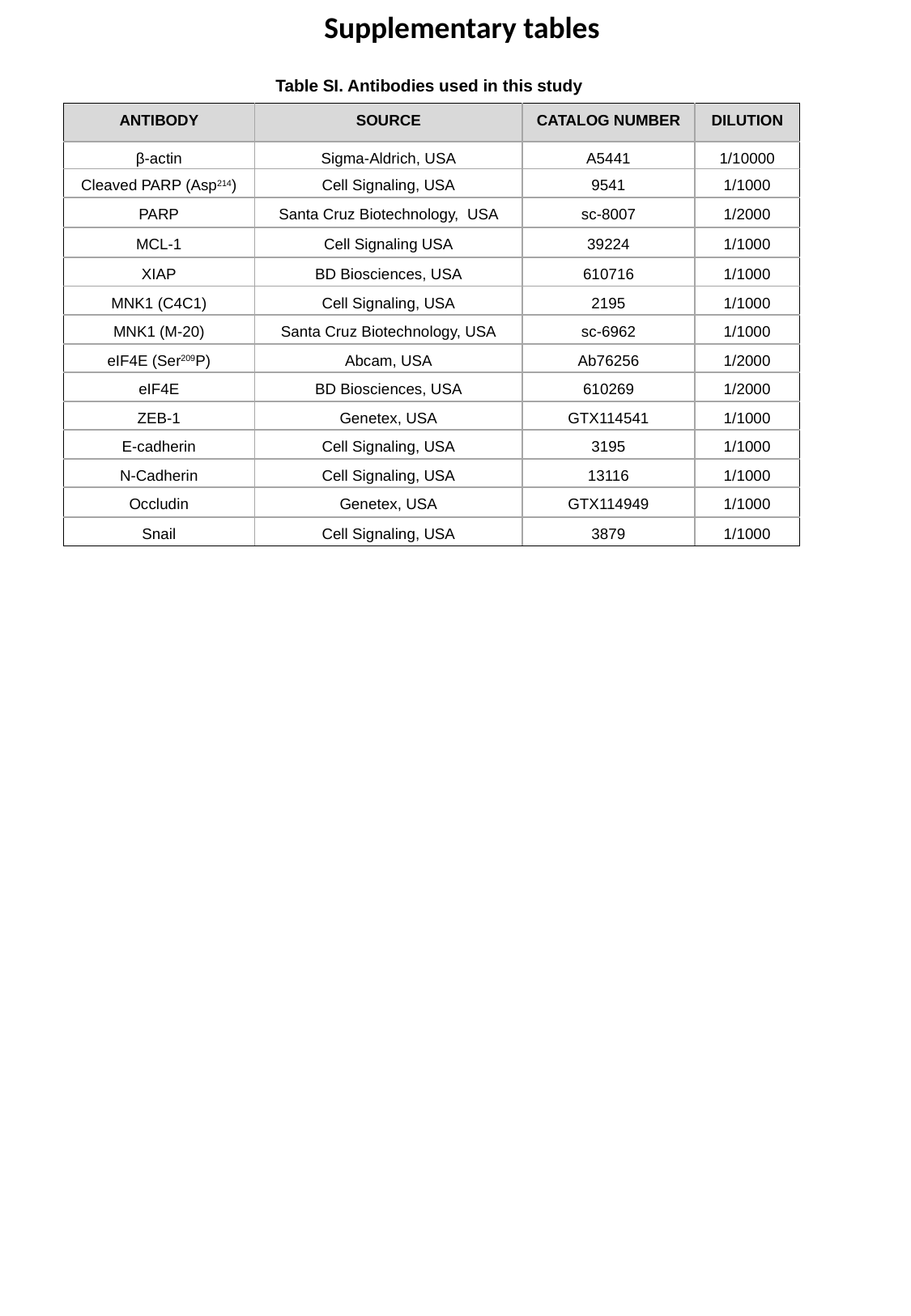

Supplementary tables
Table SI. Antibodies used in this study
| ANTIBODY | SOURCE | CATALOG NUMBER | DILUTION |
| --- | --- | --- | --- |
| β-actin | Sigma-Aldrich, USA | A5441 | 1/10000 |
| Cleaved PARP (Asp214) | Cell Signaling, USA | 9541 | 1/1000 |
| PARP | Santa Cruz Biotechnology, USA | sc-8007 | 1/2000 |
| MCL-1 | Cell Signaling USA | 39224 | 1/1000 |
| XIAP | BD Biosciences, USA | 610716 | 1/1000 |
| MNK1 (C4C1) | Cell Signaling, USA | 2195 | 1/1000 |
| MNK1 (M-20) | Santa Cruz Biotechnology, USA | sc-6962 | 1/1000 |
| eIF4E (Ser209P) | Abcam, USA | Ab76256 | 1/2000 |
| eIF4E | BD Biosciences, USA | 610269 | 1/2000 |
| ZEB-1 | Genetex, USA | GTX114541 | 1/1000 |
| E-cadherin | Cell Signaling, USA | 3195 | 1/1000 |
| N-Cadherin | Cell Signaling, USA | 13116 | 1/1000 |
| Occludin | Genetex, USA | GTX114949 | 1/1000 |
| Snail | Cell Signaling, USA | 3879 | 1/1000 |

## Slide 7
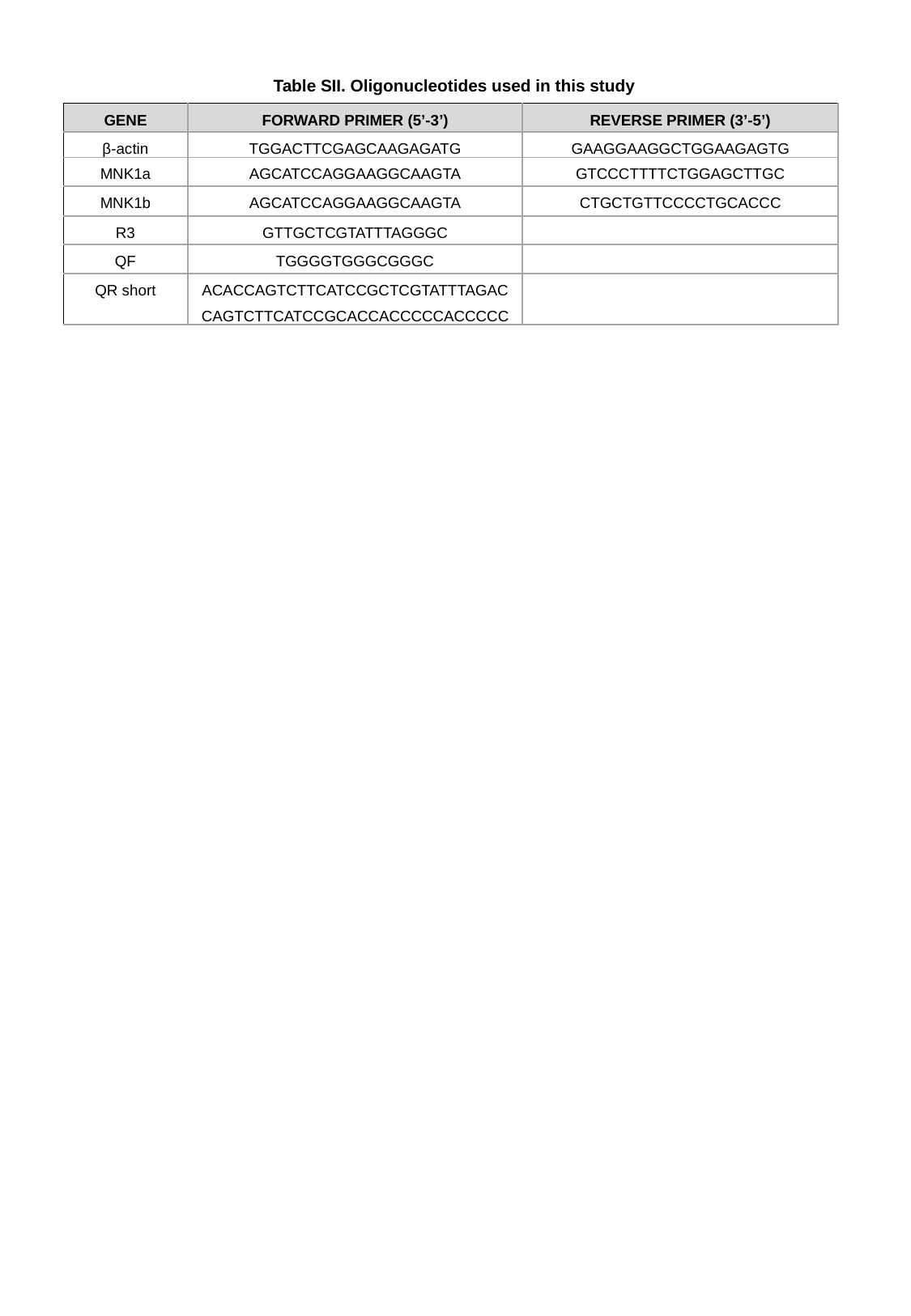

Table SII. Oligonucleotides used in this study
| GENE | FORWARD PRIMER (5’-3’) | REVERSE PRIMER (3’-5’) |
| --- | --- | --- |
| β-actin | TGGACTTCGAGCAAGAGATG | GAAGGAAGGCTGGAAGAGTG |
| MNK1a | AGCATCCAGGAAGGCAAGTA | GTCCCTTTTCTGGAGCTTGC |
| MNK1b | AGCATCCAGGAAGGCAAGTA | CTGCTGTTCCCCTGCACCC |
| R3 | GTTGCTCGTATTTAGGGC | |
| QF | TGGGGTGGGCGGGC | |
| QR short | ACACCAGTCTTCATCCGCTCGTATTTAGACCAGTCTTCATCCGCACCACCCCCACCCCC | |

## Slide 8
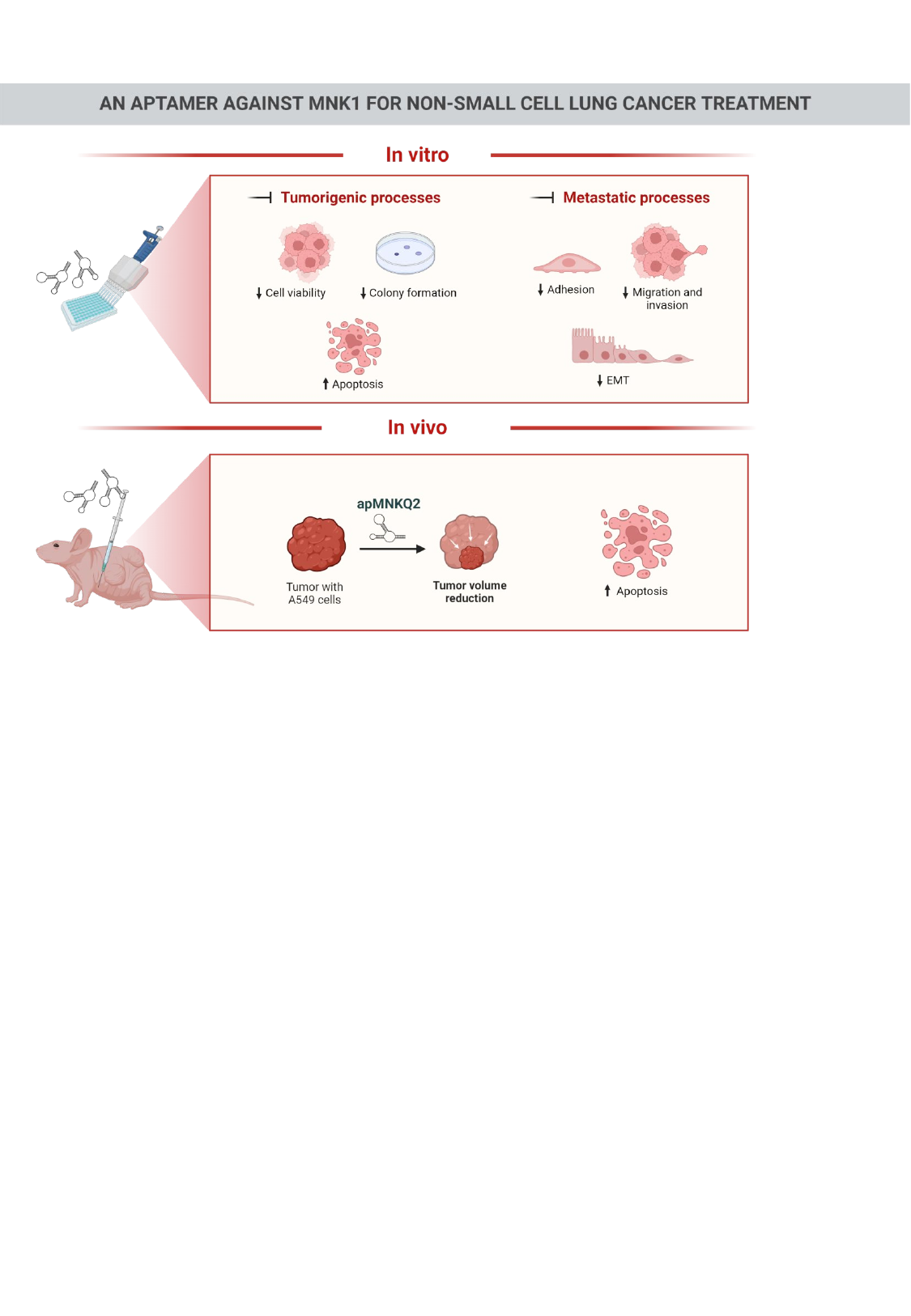

Supplement: Supplementary file 1 [file pharmaceutics-15-01273-s001.zip › pharmaceutics-2290921-supplementary-for conv/Supplementary Figures_R2.pptx]
